# A Unique Malformation of the Urethra, Associated with Piles

**Published:** 1895-04

**Authors:** Napoleon J. Bloomfield

**Affiliations:** Ohio


BLOOMFIELD, NAPOLEON J., OHIO: A UNIQUE MALFORMATION OF THE URETHRA ASSOCIATED WITH PILES. (*Columbus Medical Journal.*) Patient was thirty-five years of age. Examination found a complete absence of the penis, except a small glans, about as large as the end of the little finger, and not more than half an inch in length. There was entire absence of meatus, or urethral opening. Surrounding the glans was a narrow fold of skin, less than one-eighth of an inch in width, resembling somewhat a rudimentary prepuce. On rubbing or pinching the glans, there seemed to be an entire absence of that peculiar sensibility found in the normal organ. Just beneath and under the arch of the pubes, could be felt a small oval body, about as large as an almond. Gently rubbing this body excited the sexual orgasm, the semen passing into the rectum. The testicles were large and perfectly formed; the spermatic cords, normal. Patient had been passing urine very frequently, for three or four months, with great amount of pain and tenesmus.

Examination of the rectum showed two pile tumors on the anterior wall. About an inch and a half above the anus, just above the pile tumors, the urethra opened into the rectum. It took a strong effort to empty his bladder, the piles protruding on account of the straining; this blocked up the anus, causing spasm of the sphincter ani, dragging down the orifice of the urethra, still further interfering with the act of urination. As the tumors were so near the orifice of the urethra, and as the rectum was constantly drenched with urine, the operation of removing them was deemed inadvisable. This opinion was concurred in by Dr. Mathews, who saw the patient some time later, but he advised a thorough divulsion of the sphincter, which was done. Considerable relief resulted from this operation. The patient died from uremia.

